# The neurobiological basis of emotions and their connection to facial expressions in non-human mammals: insights in nonverbal communication

**DOI:** 10.3389/fvets.2025.1541615

**Published:** 2025-03-07

**Authors:** Daniel Mota-Rojas, Alexandra L. Whittaker, Cécile Bienboire-Frosini, Jhon Buenhombre, Patricia Mora-Medina, Adriana Domínguez-Oliva, Julio Martínez-Burnes, Ismael Hernández-Avalos, Adriana Olmos-Hernández, Antonio Verduzco-Mendoza, Alejandro Casas-Alvarado, Karina Lezama-García, Temple Grandin

**Affiliations:** ^1^Neurophysiology, Behavior and Animal Welfare Assessment, DPAA, Universidad Autónoma Metropolitana (UAM), Mexico City, Mexico; ^2^School of Animal and Veterinary Sciences, University of Adelaide, Roseworthy Campus, Roseworthy, SA, Australia; ^3^EPLFPA-Avignon, Site Agroparc, Avignon, France; ^4^Faculty of Agricultural Sciences, Animal Welfare and Ethology Specialization, Fundación Universitaria Agraria de Colombia – UNIAGRARIA, Bogotá, Colombia; ^5^Facultad de Estudios Superiores Cuautitlán, Universidad Nacional Autónoma de México (UNAM), Cuautitlán, Mexico; ^6^Instituto de Ecología Aplicada, Facultad de Medicina Veterinaria y Zootecnia, Universidad Autónoma de Tamaulipas, Victoria, Mexico; ^7^Division of Biotechnology-Bioterio and Experimental Surgery, Instituto Nacional de Rehabilitación Luis Guillermo Ibarra Ibarra (INR-LGII), Mexico City, Mexico; ^8^Department of Animal Sciences, Colorado State University, Fort Collins, CO, United States

**Keywords:** AnimalFACS, animal behavior, animal welfare, emotions, facial expressions

## Abstract

Recognizing that nonhuman animals are sentient beings has increased interest in studying their emotional state. Similar to humans, research has shown that some nonhuman mammals can modify facial expressions by contraction/relaxation of facial muscles according to their affective state. From a neurophysiological perspective, emotions are processed in several brain structures, mainly from the limbic system, such as the hypothalamus, hypophysis, hippocampus, prefrontal cortex, and amygdala. The converged pathways between the amygdala, the motor cortex, and its projections to the facial nerve control the movement of facial or mimetic muscles. Thus, facial expression is suggested to reflect the internal emotional state and could serve as an essential mode of nonverbal communication in mammals. In humans, the Facial Action Coding System (FACS) is a method that objectively analyzes facial movements using an anatomical base. In veterinary medicine, AnimalFACS is an adaptation of this system to eight animal species, including domestic animals (dogs, cats, and horses) and nonhuman primates (chimpanzees, orangutans, gibbons, macaques, and common marmosets). Considering these coded facial movements, current research aims to associate certain facial expressions with the animals’ emotional states and affective contexts. Thus, this review aims to discuss recent findings associated with the neurobiology of emotions and facial expressions in non-human mammals, using AnimalFACS to understand nonverbal communication. Characterizing each facial expression according to different contexts might help identify if the animal is expressing a positive or negative emotional response to the event, which can improve nonverbal human-animal communication.

## 1 Introduction

Human and non-human animals use facial displays to facilitate communication, encourage social interactions, and provide insight into the motivation and intention of the individual (1–4). Facial expressions are considered a less elaborate non-verbal language that might reflect the internal and external state of animals (5), as mentioned by Darwin (6), who recognized that, similarly to humans, animal facial expression changes according to negative or positive social contexts or stimuli (1, 2).

Emotions are complex reactions that allow individuals to cope with important positive and negative events that involve specific neurophysiological responses, depending on the type of stimulus experienced and the context in which the individual finds himself (7). Studying the phenomena of emotional reactions requires examining the limbic system, the network of brain structures that react to certain types of stimuli in the environment by producing emotional responses like fear, happiness, anger, or sadness (7). The limbic system comprises interconnected cortical and subcortical structures, such as the amygdala, hypothalamus, hippocampus, and prefrontal cortex, and it is central to emotional processing in mammals (8). It offers communication between visceral states, from emotion to cognition and behavior (9), while acting as a control center for emotions, behavior, and memory (10).

Emotions serve as a key component of survival, influencing behavior, decision-making, and social interactions across various species. Hence, animal emotions are intertwined with animal welfare, and accurate assessment of animal emotions is crucial in animal welfare research (11). Animal welfare issues can be assessed by studying and understanding the emotions they experience and how they express them bodily (12). Although various authors have studied facial expressions in different species (13–18), their role in expressing emotions is still controversial. For this reason, interest has recently increased in studying animals’ emotions and how they can be conveyed through facial movements (19). In this sense, facial expressions have been used to identify animal pain and assess its severity (8, 20). They have been demonstrated to encode the sensory and affective components in humans suffering pain (9). Observing that in non-verbal humans, doctors used scales to evaluate pain perception and severity, Langford et al. (10) applied these scales in mice, separating the typical sensory response from the emotional response to painful stimuli by lesioning the insula. This started a new field of investigation, interpreting emotions through facial expressions in non-human animals.

The study of facial expressions considers the neurobiological basis of the motor control of facial muscles (11, 12, 14), with the determinants that elicit the change (e.g., an encounter with a predator or unfamiliar conspecific) and the meaning of said facial expression (e.g., aggression or play) (15–17). The first approach to studying facial expression in humans was developed by Paul Ekman and collaborators (21) through a comprehensive and anatomically based system called the Facial Action Coding System (FACS). FACS is a standardized coding system that describes visible facial movements or action units (AU) according to facial or mimetic muscles (12, 19, 22–24). In humans, a facial expression of happiness is codified as the combination of the AU 6 + 12 (cheek raiser and lip corner puller). Thus, FACS associates each AU with the underlying muscle; for example, the inner brow raiser (AU1) with the frontalis pars medialis muscle.

In veterinary medicine, human FACS was used as a reference to adapt coding systems in animals, and it is called the AnimalFACS. The first AnimalFACS was developed in chimpanzees (*Pan troglodytes*) (ChimpFACS) due to their anatomical resemblance with humans (11). To date, eight validated AnimalFACS have been published for rhesus monkeys (*Macaca mulatta*) (MaqFACS) (25), gibbons (*Symphalangus syndactylus, Hylobates pileatus, Hylobates moloch, Nomascus siki, N. gabriellae, N leucogenys, H. muelleri*) (GibbonFACS) (16), orangutans (*Pongo* spp.) (OrangFACS) (26), dogs (*Canis lupus familiaris*) (DogFACS) (27), cats (*Felis catus*) (CatFACS) (28), horses (*Equus caballus*) (EquiFACS) (29), and common marmosets (*Callithrix jacchus*) (CalliFACS) (30). The present review will focus on the discussion of these species.

Although AnimalFACS describe specific AUs according to the species (15), unlike humans, studies associating certain AUs with an emotional valence are limited. The neurobiological bases of basic emotions are well-defined in humans and share remarkable similarities with animals (31–33). However, the neural pathways comprising the control of facial expressions during emotional management are poorly understood, and studies employing FACS during brain mapping are limited. Even so, it has been possible to reveal the coordinated participation of organized sections of the primary motor cortex, the facial motor center (VII), and emotional centers such as the amygdala (34, 35). Facial expressions are controlled by the facial nucleus, located in the brainstem, which sends motor signals to the muscles of the face. In mammals, the facial nucleus receives input from various brain regions, including the limbic system and motor cortex, allowing emotional states to influence facial muscle activity directly (36). In this way, these connections could command the mechanism of action that promotes facial expressions during an emotion. This review discusses recent studies on the connection between the neurobiology of emotions and facial expressions in non-human mammals and their significance in understanding and improvement of nonverbal communication.

## 2 Search methodology

The databases PubMed and Web of Science were used to search for adequate papers. The following keywords were used alone or in combination to perform the search: “animal FACS,” “animal emotion,” “neurobiology of emotion,” “emotional valence,” “animal facial expression,” “ChimpFACS,” “MaqFACS,” “OrangFACS,” “GibbonFACS,” “DogFACS,” “EquiFACS,” and “CalliFACS.” The inclusion criteria were papers discussing the neurobiology of emotion –in both humans and non-human animals–, papers focusing on animal emotions, those addressing the neural control of facial expression in mammals, and those where validated AnimalFACS were used in positive and negative contexts. There was no selected date for the papers, and the search was performed in English and Spanish. Papers that discussed animal facial expressions but did not have a validated AnimalFACS were excluded from this review.

## 3 Neurobiology of positive and negative emotions in animals

Animal welfare includes the absence of negative states but also the presence of positive states (37–39). Research done by Jaak Panksepp clearly shows that animals have both negative and positive emotions, including the seven basic emotional circuits: FEAR, RAGE (anger), PANIC (separation anxiety), SEEK (motivation to explore), LUST (sex), NURTURE (social bonding), and PLAY (40). Fear, rage, and panic are considered negative emotions, while seek, lust, nurture, and play are positive ones (41). Emotions represent adaptive brain states of organic regulation that have been conserved throughout evolution (2, 12, 42, 43). Studies have shown that animals have positive and negative emotional systems (40, 44).

**Figure 1:**
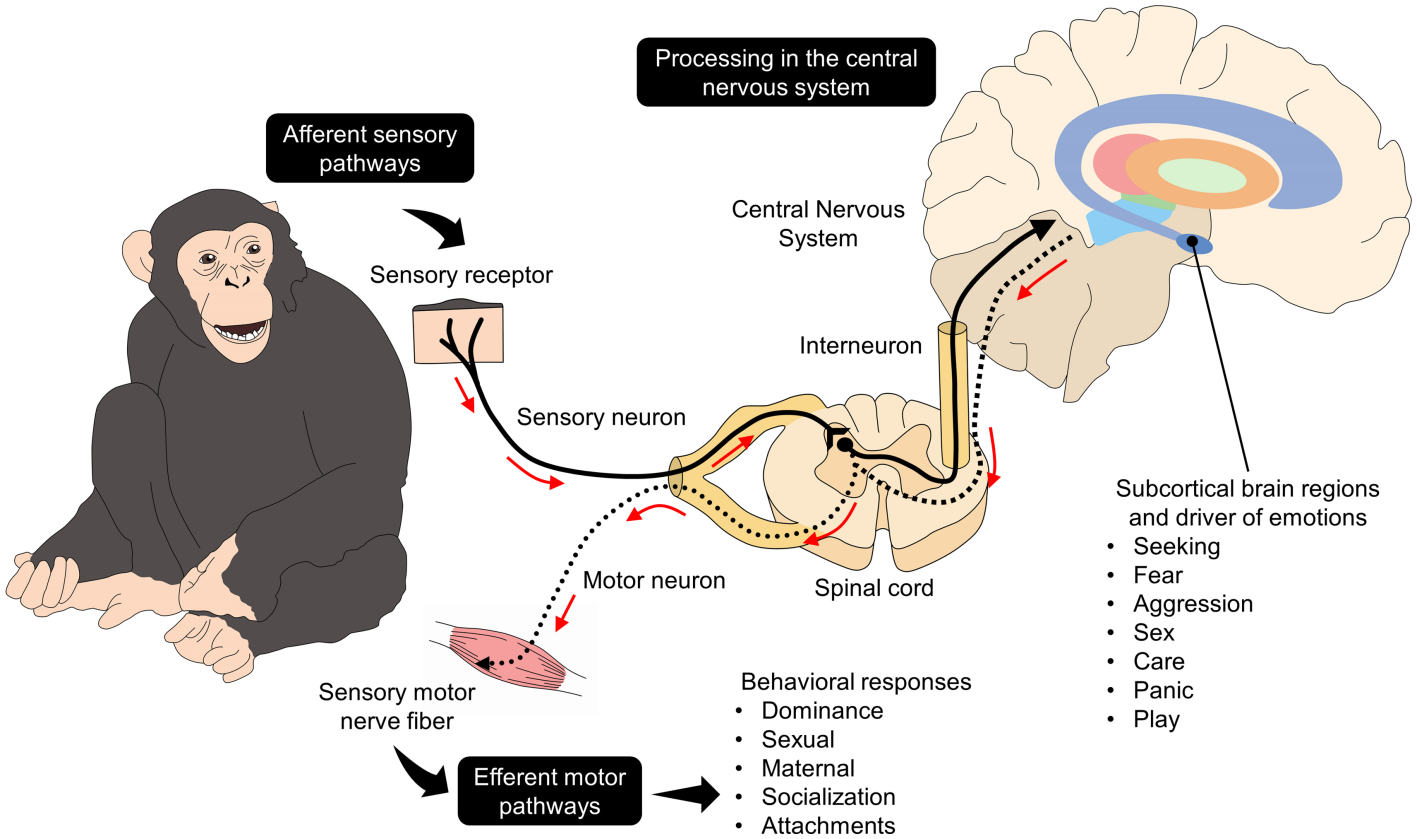
Neurobiological pathways of behavior linked to their affective state. The brain depicts some brain regions related to emotional processing, such as the striatum (purple), globus pallidus externus (orange), globus pallidus internus (light green), thalamus (red), substantia nigra (green), hypothalamus (light blue), amygdala (dark blue).

The neurobiology of behavior responds to several stimuli that need to be processed in the central nervous system and subcortical structures basic structures such as the limbic system, which is widely related to emotional management since it houses higher brain centers with extensive and ubiquitous networks such as the amygdala, thalamus, hypothalamus, hippocampus, prefrontal cortex, among others (45–49). As schematized in Figure 1 (40, 50), afferent pathways project internal and external stimuli such as hormone levels or information arising from environmental conditions from the periphery to the spinal sensory neurons (51). The interneurons in the spinal cord project the stimulus to subcortical regions and cortical brain structures that regulate behavior’s motor and affective aspects (including facial expression). Furthermore, subcortical structures such as the hypothalamus modulate the endocrine and physiological changes (e.g., tachycardia, cortisol increases) associated with behavioral responses in specific contexts (48, 50).

The amygdala is the leading center of the limbic system, where the emotional response to several contexts is integrated. However, although most studies focus on negative emotional responses (52, 53). For example, studies on rodents have demonstrated that the amygdala activates in response to threatening stimuli and plays a key role in coordinating defensive behaviors, including facial expressions of fear or aggression (52). In the same species, Lee et al. (54) found an increased neuronal electrical intensity in the basolateral region when exposing mice to conditioning, a response accompanied by behavioral changes related to emotional valence. In mice, Kennedy et al. (55) demonstrated that electrical neuronal activity and neurotransmitter density in the extracellular matrix of the hypothalamic dorsomedial and ventromedial subdivisions increase and persist in response to the presence of a predator.

**Figure 2:**
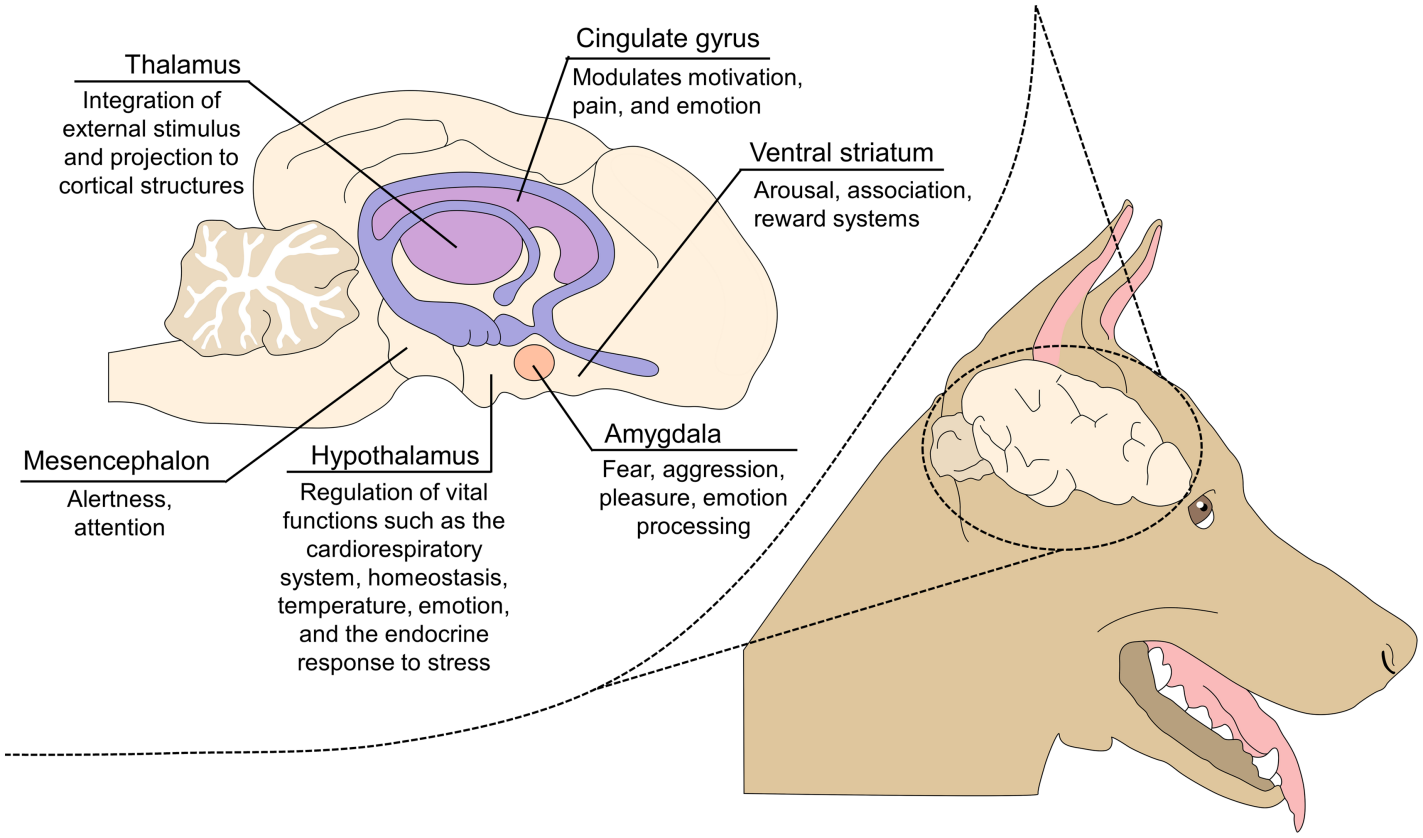
The limbic system and its role in emotional processing.

In primates, damage to the amygdala impairs the ability to recognize emotional facial expressions in conspecifics, emphasizing its role in producing and interpreting emotional signals (56). Similarly, Chou et al. (53) evaluated the effect of simulated dermal stimulation through transcranial-focused ultrasound on the activation level of the amygdala and coordinated circuits (e.g., the anterior cingulate cortex, the hippocampus, and the prefrontal cortex). The authors found a positive correlation between lower amygdala activity and the absence of simulated anxiety and fear:, However, the connectivity with other regions was limited. Figure 2 describes the main structures of the limbic system that participate in the development of behavioral manifestations and emotional processing (57).

During conditioned fear, Haubensak et al. (58) found modifications in the electrical activity of the basolateral region accompanied by the absence or decrease in the expression of imminent behaviors such as freezing. Likewise, it is well documented that risk analysis of a threat is processed by the synchronized activity of the macrocircuit composed of the bed nucleus of the stria terminalis (BSNT), ventral hippocampus (vHPC), ventromedial prefrontal cortex (vmPFC), basolateral amygdala (55, 59–61), periaqueductal gray (PAG) (62), *Locus coerelus* (LC) (63), parabrachial nucleus, posterior insular cortex (64), *nucleus accumbens*, *ventral pallidum* (65), among others. The cingulate cortex-hippocampus-basolateral amygdala is considered one of the main pathways to process generalized fear of unknown environments (66).

Regarding positive emotions, affective neuroscience suggests several limbic and extra-limbic structures process them. For example, pleasure is processed by the so-called hedonic sites, including the orbitofrontal or prefrontal cortex, the *nucleus accumbens*, the insular cortex, the anterior cingulate cortex, the *ventral pallidum,* and the amygdala. In humans, rats, and orangutans, these regions become warm spots with pleasant flavors during palate stimulation. These changes are accompanied by changes in facial expressions (67). However, little is known about the organization of these structures to build and integrate a positive emotion from a pleasant sensory stimulus (68).

Therefore, it is important to find ways to generate behaviors that cause happiness instead of focusing exclusively on behaviors related to needs that must be satisfied to avoid suffering (31–33, 37). A possible explanation for why there has been more interest in investigating negative emotions over positive ones is that the procedures that produce suffering result in negative effects on animal welfare, a major source of concern for the public (34). Furthermore, the absence of signs of pleasure or positive affect may indicate states of emotional distress (35, 36).

Broom (31) mentions that impossible to determine the feelings of any individual or species accurately and that measuring animal emotion is challenging because they cannot verbally express what they feel (69). Therefore, it is evident that the scientific study of emotional states requires the development of precise measurement instruments. In particular, finding new meaningful, practical, reliable, positive welfare indicators, represents an important challenge in animal welfare science nowadays (70, 71). For example, different parameters of good welfare have been proposed and are currently being studied (38, 72).

Dawkins (73) mentioned that “behavior is of crucial importance in gauging what an animal wants.” Various studies describe the interest in vocalizations (74, 75), tail movements (75–77), nest-building (78), play (79), self-grooming (38), or anticipatory behavior (80). Cognitive bias, especially judgment bias, has also been proposed to assess positive emotions (81). Applied ethology studies a wide range of animal species with different emotional repertoires and different behavioral patterns. Another challenge is to describe the range of positive emotions in each animal species and to include all species regardless of their zootechnical purpose, from those used in laboratories, zoos, companion animals, and raised in farms (38).

The autonomic nervous system, particularly the activity and balance of the sympathetic and parasympathetic branches, through Heart Rate Variability analysis, has also been linked to positive emotions such as appreciation in humans (82) and could be used similarly in animals (83–86). Furthermore, neurobiology studies with assessments of brain and/or neuroendocrine system activation offer promising prospects for positive welfare assessment (87–89): imaging techniques (90, 91) and measures of neurotransmitters/neuropeptides (such as dopamine, opioids, and oxytocin) could provide interesting information (38, 76, 88, 92) to evaluate positive events. The latter is largely underexplored. In this sense, in a study conducted by Levenson et al. (21) in humans, differences were found in the physiological parameters evaluated when comparing the most common negative (fear, anger, and disgust) and positive emotions (happiness and surprise). For example, anger and fear increased heart rate more than sadness. Temperature increased in the presence of happiness and decreased in fear. The authors also found differences between negative emotions only in the facial action task directed at anger. On the other hand, fear and sadness produced cardiomegaly, increasing the presentation rate in cases where the main emotion was disgust, causing a decrease in heart rate (21).

## 4 Is pain an emotion?

It is widely recognized that pain has an emotional impact on both humans and animals. Pain is “an unpleasant sensory and emotional experience associated with, or resembling that associated with actual or potential tissue damage” (93). Although this definition does not exclusively refer to animals, it implies that painful stimuli for humans will also cause pain in other species (94).

Pain has often been considered an emotion due to its relation to other negative states, such as fear and anxiety (95). Authors such as Monteiro et al. (96) mention that pain causes negative emotions in animals, such as fear, anxiety, and frustration. Thus, it is necessary to consider promoting positive experiences to reduce pain when managing this sign. Promoting positive experiences is not only related to pain but also to improving species-specific behaviors and quality of life. An example of this effect has been observed in human medicine, where the integration of psychological support can reduce maladaptive pain sensation levels (97, 98).

The possible explanation of emotions’ negative modulation effect on pain is due to their shared neurobiological pathway (99, 100). When the spinothalamic and spinoreticular tracts project nociceptive stimuli, they are processed in higher cerebral structures, mainly the cerebral cortex, where intensity and direction are defined (101–103). The amygdala also participates in this process by projecting fibers that coordinate the functioning of the cerebral cortex, especially the primary motor cortex (104). Interestingly, the amygdala in its basolateral region is mainly responsible for processing emotional pain and facial expression information due to the presence of adrenergic and oxygenic fibers that interconnect with regions such as the cerebral cortex, somatosensory, and primary motor cortex (105).

Therefore, due to the relationship between the amygdala, and other regions of the Central Nervous System, it is suggested that pain can be considered an emotion. Strobel et al. (106) emphasize that the amygdala in its basolateral portion emits adrenergic fibers to the primary motor cortex, generating motor responses. Moreover, pain is a sensory characteristic and a personal experience influenced by biological, psychological, and social factors (93).

Although the neurobiological basis may suggest that pain can be considered an emotional experience in animals, evidence is needed to support this idea. The results reported by Nakashima et al. (107) might help to improve this perspective. These authors examined whether a painful emotional experience influenced the sensitivity of emotional expression recognition in 60 naïve male Long-Evans rats. The animals were exposed to four phases: the baseline preference test, pain manipulation test, post-manipulation preference test, and state anxiety test. When pictures of pain or neutral expressions were shown, the animals more frequently entered boxes with neutral expressions compared to pictures of pain (time of entries neutral expressions = 11 vs. time of entries pain expressions = 8). In addition, no differences were observed between groups of animals in the anxiety test. These results suggest that animals can recognize pain through facial expressions. Indeed, Hadj-Bouziane et al. (108) evaluated the effects of macaque facial expressions on neural activation within these two regions using functional magnetic resonance in three awake monkeys. They observed that exposure to four different facial expressions (neutral, aggressive, fearful, and submissive) caused activation of the temporal cortex and the amygdala, specifically the dorsal part of the lateral nucleus. Thus, the results presented by both authors suggest that animals recognize changes in the facial expressions of conspecifics and can respond to them, which is directly coordinated by the amygdala.

The knowledge derived from an animal’s facial expression associated with pain could be evidence that pain is an emotional state (109). In large ruminants and equines, the typical facial expression of pain comprises asymmetrical ears, orbital tightening, tension of the muzzle, mimic muscle, and nostrils (20, 110, 111). However, these facial changes are markedly different from other movements occurring in any emotional state (2, 42). That is, the AUs recorded in a “pain face” are not described within the FACS, perhaps because this system details the natural contraction of the muscles of the face (23, 112). Thus, since changes in the facial expression during pain perception are unique, considering pain as an emotion would be limited. Pain has a multidimensional nature where sensory, neurobiological, social, and even emotional aspects must be considered for each species and each individual (42, 107, 113).

## 5 The role of facial expressions in non-verbal communication of non-human mammals

Darwin’s thesis (6) was the first document to argue that, just like in humans, the emotional state of animals can modify their body language due to their sentience (37). However, the role of facial expressions was still researched as a means of communication in animals. According to Waller and Michelleta (5), emotions are an inflexible expression of the internal and external state of animals’ internal and external states. This state can be conveyed through non-verbal language including body postures and facial expressions.

Nonhuman primates are a perfect example of how facial expressions can be used to communicate with conspecifics according to the context (e.g., playful or agonistic) (15, 16). In great apes, the so-called play face or open mouth face —mouth completely open with fully exposed canine teeth and palate— is observed in positive contexts, invitating to initiate play bouts with conspecifics (114) or during gentle play (115). In gorillas, Tanner and Byrne (116) have reported that playful interactions can start as soon as 4 s after a gorilla displays a play face.

Similarly, a variant of the play face, the full play face —open and relaxed mouth with complete exposure of the upper and lower teeth— is another example where facial expression is a communication channel between nonhuman primates, particularly during play fighting (117–120). Nonhuman primates also display more facial expressions when the recipient is visually attentive to other animal faces (121). This is the so-called audience effect, where species like siamangs exclusively perform the mouth-open half, mouth-open full, grin, and pull a face when interacting with conspecifics (122). Similarly, Waller et al. (123) found that orangutans have more intense and complex facial displays when the recipient –a conspecific– is highly attentive, showing that facial expressions are important to convey intentionality.

The recipient effect on the presentation and frequency of certain facial movements can also be observed during interspecific interactions, as reported in human-dog dyads (1). In contrast to wolves, domestic dogs have the AU101, which is responsible for the contraction of the *levator anguli oculi medialis* and *retractor anguli oculi lateralis* muscles to raise the eyebrows (124). This difference gave the dog an advantage in improving its interaction with humans, suggesting the role of facial expression as a means to facilitate interaction with other species. In this sense, Kaminski et al. (125) reported that the frequency of the facial movement known as inner brow raiser (AU101) increased when humans were attentive to the dogs’ presence (frequency of AU101 in attention = 0.14 vs. no attention = 0.05), while non-social stimuli did not affect this facial movement.

The so-called facial mobility hypothesis is another instance where facial expression is related to a species’ communicative repertoire, as mentioned by Florkiewicz et al. (126) in chimpanzees and gibbons. These authors concluded that species with more complex socio-ecological environments (e.g., chimpanzees) have higher facial mobility and display more AU combinations as an evolutive trait.

This evolutionary perspective suggests that some species use less elaborate behaviors that involve less energy expenditure, such as changes in body posture, to communicate emotional states. For example, in domestic or wild canines, lip lifting with full exposure of the fangs serves as a warning signal to avoid conflict (1). Similarly, Camerlink et al. (127) reported that pigs use facial expressions to signal intention and emotional state. When evaluating 38 pigs during agonistic interactions, significant differences in the ear angle, snout ratio, and eye ratio were reported. It was found that during aggression, the animals’ ears were frequently positioned forward. In contrast, during withdrawal, the ears were positioned backward (*p* < 0.0001). Furthermore, the eye ratio was larger in aggression-inciting animals compared to non-aggression-inciting pigs (1.05 + −0.03 vs. 0.99 + −0.03, *p* = 0.04), in addition to decreased snout proportion.

This is a clear example that animals modify their facial expression to indicate an emotional state and the intention of said state, which is necessary for highly social species. In horses, facial expression, behavior, and locomotion were evaluated by Phelipon et al. (128) under two conditions: presenting a bucket of food (positive valance). During this event, the horses exhibited changes such as a lower neck position with ears forward and upper lip advanced. They went faster by increasing their stride frequency, which was accompanied by increased global locomotor activity. The exposure of the horses to food was available without allowing them to eat generated the animals exhibiting a higher neck position with the ears backward or to the side, accompanied by ear movements and eye blinks. Both results agree that animals can generate changes in their facial expression based on the emotion they are experiencing. In cats, ear flattening or bristling are changes related to negative emotional states such as fear (42). Thus, facial expressions facilitate social interactions between conspecifics by conveying the sender’s intention.

In equines, Lundblad et al. (129) observed significant changes in the facial expression and physiological parameters of 28 healthy horses during transport and social isolation. During transport, considered a stressful event for animals, an expected increase in heart rate was observed (between 25 and 35 beats per minute). Moreover, the frequency of AUs such as ear blink, white eye show (AD1), tongue show (AD19), nostril dilator (ED38), lip part (AU25), upper eyelid elevator (AU5), internal brow elevator (EAD101) and ear rotator (EAD104) increased by 20% compared to baseline. These results suggest that when faced with adverse events, the stress response involves the physiological function of animals and their facial expression (50).

Animals are social and emotional species that rely on different pathways to interact and communicate with conspecifics and other species. By examining the facial expressions of other animals, the recipient can understand the emotional state or the sender’s intention. Thus, certain species, such as monkeys, intensely focus on eye and mouth movements to judge facial expressions (130). Hence, these results suggest that the function of facial expressions might depend on the species and the complexity of the interrelations.

## 6 FACS applied to study animal’s facial expressions: currently published AnimalFACS

The association between an emotional state and changes in facial expression has led to the development of coding systems describing facial movements, a facial expression made from several muscular movements, and their possible association with an affective state. FACS is a methodology developed by Ekman and Friesen (22) that describes facial movements based on the anatomical contraction of mimetic muscles. FACS designs an alphanumeric code for each AU (e.g., AU101 refers to the inner lip raise due to the contraction of the *levator anguli oculi medialis* and *retractor anguli oculi lateralis muscles*) (27, 131). Although FACS was initially designed for humans, this system has been adapted to different species, which will be discussed below.

### 6.1 FACS in nonhuman primates: the first approach to an animal-related study of facial expression

Similarly to humans, nonhuman primates highly rely on different facial expressions to communicate and maintain social networks with conspecifics (15, 16). In particular, chimpanzees are the closest species to humans and share 16 underlying mimetic muscles such as the *frontalis, orbicularis oculi, levator labii superioris, zygomatic major*, among others (19, 132, 133). Due to this resemblance, ChimpFACS was the first AnimalFACS developed in chimpanzees (ChimpFACS) by Parr et al. (11). Through dissection of the mimetic muscles, the authors described 15 AUs with an equivalent muscular origin to humans (11). However, the AU160 (the lower lip depressor) is a facial movement exclusively described in chimpanzees (11, 19).

Regardless of the similarities, studies highlight a specific difference in chimpanzees, in whom facial expression is relevant, and facial displays, together with fast vocalizations, are part of the behavioral repertoire of nonhuman primates (134, 135). This also has anatomical implications, where humans have a higher proportion of slow-twitch muscle fibers in the *zygomaticus major* (15%) and *orbicularis oris* (22%) than primates, such as chimpanzees (1 and 7%, respectively) and rhesus monkeys (5 and 7%, respectively) (135). Moreover, the same authors mention that mimetic muscles around the oral cavity of chimpanzees are thicker due to the importance of vocalization and pout faces in the species (132).

The second FACS adapted to great apes was OrangFACS, for orangutans, where Caeiro et al. (26) established 17 AUs to codify common facial displays in the species, such as the play face (AU10 + 12 + 25 + 27). However, differences are also present. While the authors found that no AUs are orangutan-exclusive, AU4 (an AU thought to be exclusive of humans) and AU18 (not seen in chimpanzees) were observed in this species (26).

In the case of gibbons and siamangs, the GibbonFACS was developed by Waller et al. (16) for hylobatids, describing 18 AUs. This coding system has shown the importance of facial expressions during social contexts in five species of gibbons. For example, Scheider et al. (121) determined that during interactions with conspecific (e.g., grooming, agonistic, and playing), gibbons displayed more facial expressions where open-mouth displays, including the AU10, AU16, AU25, AU26, and AU27 were involved (e.g., upper lip raiser, lower lip depressor, lips parted, jaw drop, mouth stretch). Additionally, Florkiewicz et al. (136) evaluated three species of hylobatids (*Nomascus*, *Hoolock*, and *Hylobates*) to establish the association between facial expressions and strengthening pair bonds. It was found that hylobatides have approximately 80 unique facial expressions and are used to promote and enhance social interactions.

**Figure 3:**
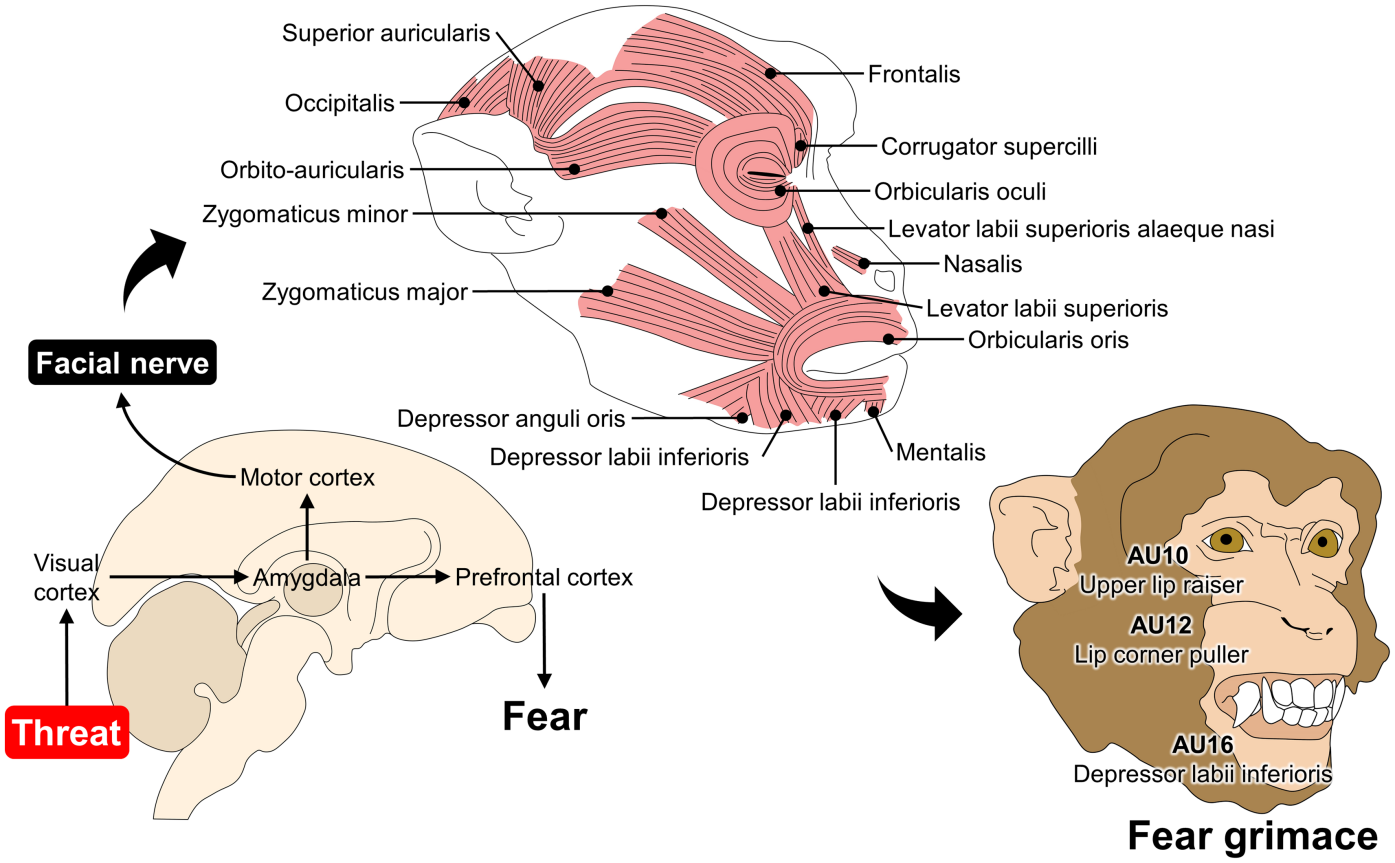
Association between fear and facial expression changes in Rhesus monkeys. When macaques perceive a threat (e.g., predator or unfamiliar conspecific), different pathways are activated to elicit fear-related responses. For example, visual inputs of a threat travel from the visual cortex to the amygdala and subsequently to the prefrontal cortex to consciously perceive fear. The amygdala also projects neurons to the motor cortex, where direct connections to the facial nerve modulate mimetic muscles. In monkeys, the fear grimace is coded as AU10 + 12 + 16, where the *levator labii superioris*, *depressor labii inferioris*, and *zygomaticus* participate in modifying the facial expression of macaques.

For monkeys, the MaqFACS for rhesus and CalliFACS for common marmosets have established 15 AUs for each species to describe their facial repertoire (25, 30). A particularity of monkeys and the MaqFACS is the description of ear movements: EAU1 (ears forward), EAU2 (ear elevator), and EAU3 (ear flattener). The ability to independently move the ears and change their position together with the facial expression is only present in this species. It has been lost in apes such as chimpanzees or humans (25, 123). For example, facial expression research has been mainly performed in rhesus monkeys. Ongoing research has developed automatic detection of facial expressions such as lip-smacking, threat, alert, and fear grimaces related to negative affective states as a response to an intruder (Figure 3) (137). Moreover, MaqFACS has also been used to perform cross-species comparisons in Barbary macaques (*Macaca sylvanus*) and Japanese macaques (*Macaca fuscata*) (123, 138). In the case of Japanese macaques, four new AUs were detected when compared with AUs for rhesus monkeys (19 AUs and 3 ear movements), such as the nose wrinkler and upper lip raiser (AU9 + 10), true pucker (AU18i), and outer pucker (AU18ii) (138) (Figure 3).

Although human and nonhuman primates share certain AUs and might cause the same visible change, it is essential to consider the context to assign a possible emotional valence or meaning to the facial expressions of animals. Detailed facial expression ethograms have been published in monkeys and great apes, where a description of the appearance and the suggested social/emotional meaning or function is included (11, 139, 140). For example, most ethograms consider the following nine facial displays: bared-teeth display, relaxed open-mouth face or play face, pant-hoot, ambiguous faces, neutral faces, scream, alert face, pout, and whimper (11).

The silent bared teeth face resembles a human smile —an open mouth with retracted corners and fully exposed teeth— and involves the movement of the *zygomaticus major* muscle (AU10 + 12 + 16 + 25) (141). However, captive chimpanzees do not exhibit this facial expression during playful interactions but as an appeasement signal when encountering subordinate and dominant individuals (3, 142). Moreover, in the case of Sumatran orangutans (*P. abelii*), Petersen and Higham (143) associated this expression with fear followed by aggression. In contrast, the play face (AU12 + 25 + 26) is analogous to a human smile and is observed in monkeys and great apes during play bouts. It requires movement from the *zygomaticus major* muscle and the contraction of the *levator labii superioris muscle* to open the lips (123, 142).

Although AnimalFACS for nonhuman primates represents 62.5% of published coding systems in animals, many primate species, such as gorillas (*Gorilla gorilla*) are still being studied. The facial expression repertoire in this species includes play faces, pouts, and bared-teeth displays (144). A validated FACS has not been published; however, Dobson (145) and Waller and Cherry (16) made a comparative approach to suggest 15 AU in gorillas and codify facial expressions such as the play face (AU16 + 25 + 26) or the full play face (AU10 + 16 + 25 + 26). Furthermore, Rotenstreich and Marom (133) recently performed detailed anatomical studies in a female gorilla, where 18 mimetic muscles were found. Some differences were also reported, such as the absence of the *risorius* muscle, in contrast to humans and chimpanzees.

In primates, studies focusing on facial expression emphasize that current AnimalFACS are not facial ethograms of the species. The presence or absence of an AU cannot be directly linked to an emotional state. However, the presentation of specific facial movements (e.g., play face) can be codified with AUs, providing an objective and anatomically based description of a facial action during a positive/negative context.

### 6.2 Updating FACS in dogs

**Figure 4:**
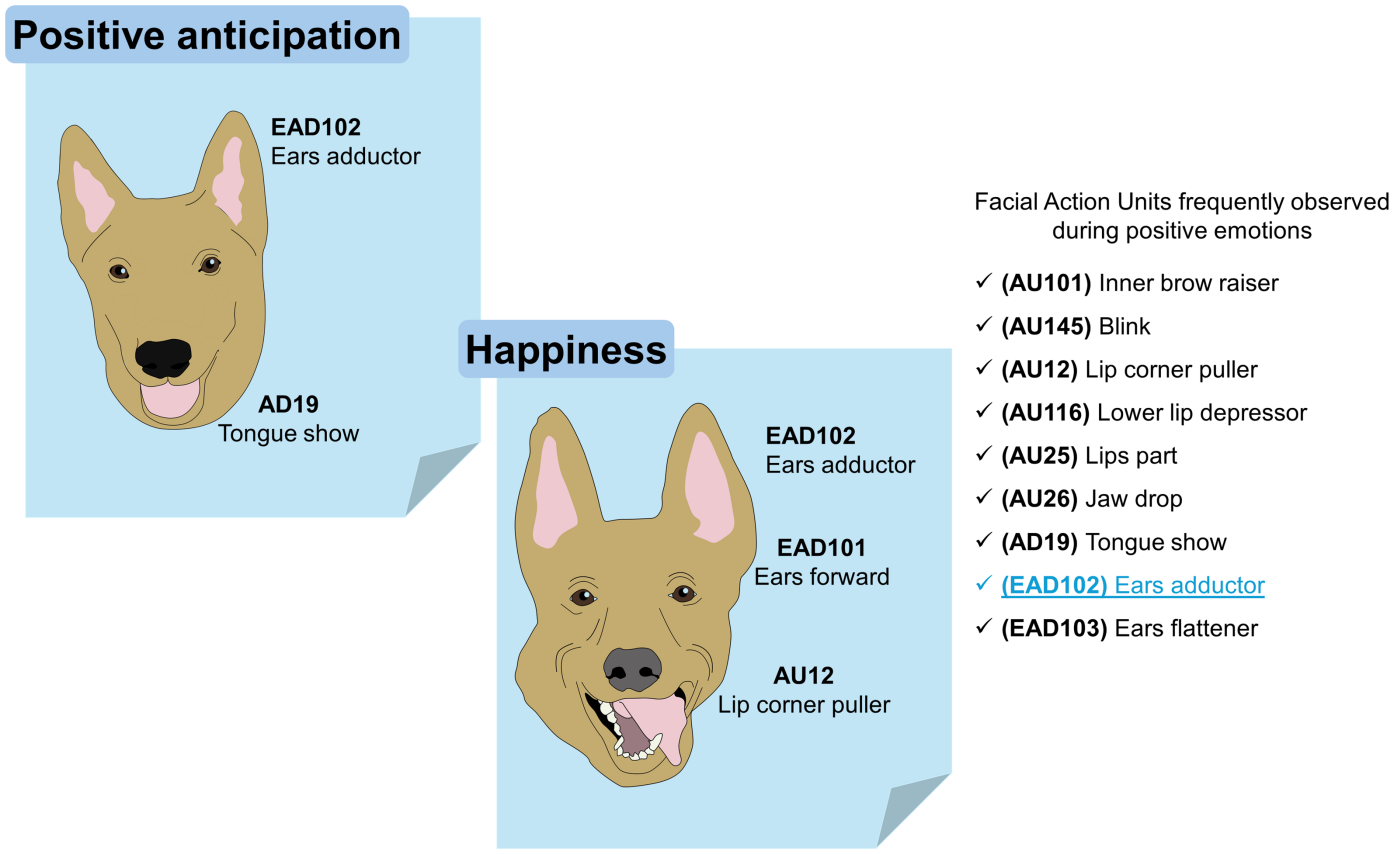
The facial expression of dogs during positive emotions. Positive anticipation and happiness are two positive emotions that have been researched in dogs. In both, the FAU observed exclusively during positive events is the EAD102 (written in blue inside the image).

Bolwing (146), has described various facial expressions in dogs that show emotions for many years. In the same way, multiple similarities have been detected between the facial expressions of dogs, primates, and humans (27), among which we can mention fear, anger, sadness, and happiness. This coincides with what was reported by Bloom and Friedman (147), who found that facial expressions observed in dogs were joy, sadness, surprise, disgust, anger, fear, and neutrality. The development of the FACS for the domestic dog (DogFACS) led to the establishment of feasible analogies with humans, with whom they interact daily (27). Both dogs and humans share a common environment. Therefore, Caeiro et al. (148), decided to test whether dogs show specific discriminatory facial movements in response to different types of emotional stimuli and whether grimaces are similar to those of humans when reacting to emotionally comparable contexts. Their results showed that dogs do not exhibit human-like facial movements in comparable emotional situations. Likewise, it has been seen that the position of the ears in these animals is strongly related to negative emotions caused by environmental stimuli (149, 150). Figure 4 schematizes the FAUs that are frequently observed in dogs during positive emotions (148, 151).

### 6.3 Updating FACS in cats

CatFACS are an adaptation developed by Caeiro et al. (152), which encodes 15 AUs and 13 diverse movement or action descriptors ADs of which only seven Ear Action Descriptors (EADs) focus on the dynamics of the auricular appendage. The difference in classification lies in the musculature. AUs are mimetic muscle movements, and the ADs code covers non-mimetic muscle actions such as movements of the ears and tongue, among others. Particularly, in the cat, the whisker muscles (*Lateralis nasi*, *Orbicularis oris*, and *Caninus*) play a transcendental role in facial expressions. That is why this adaptation of the FACS has been extensively researched in domestic cats (28, 152). This tool is a valuable resource due to aspects such as the current global distribution of the domestic cat (*Felis silvestris catus*), which positions it as the preferred companion animal (153). However, the number of reports that use it is limited. Even so, it has been possible to relate it to inter-specific human-animal interactions. For example, Bennett et al. (154) developed a validation study to detect the behavioral adjustments of cats in confinement contexts without and with human interaction.

**Figure 5:**
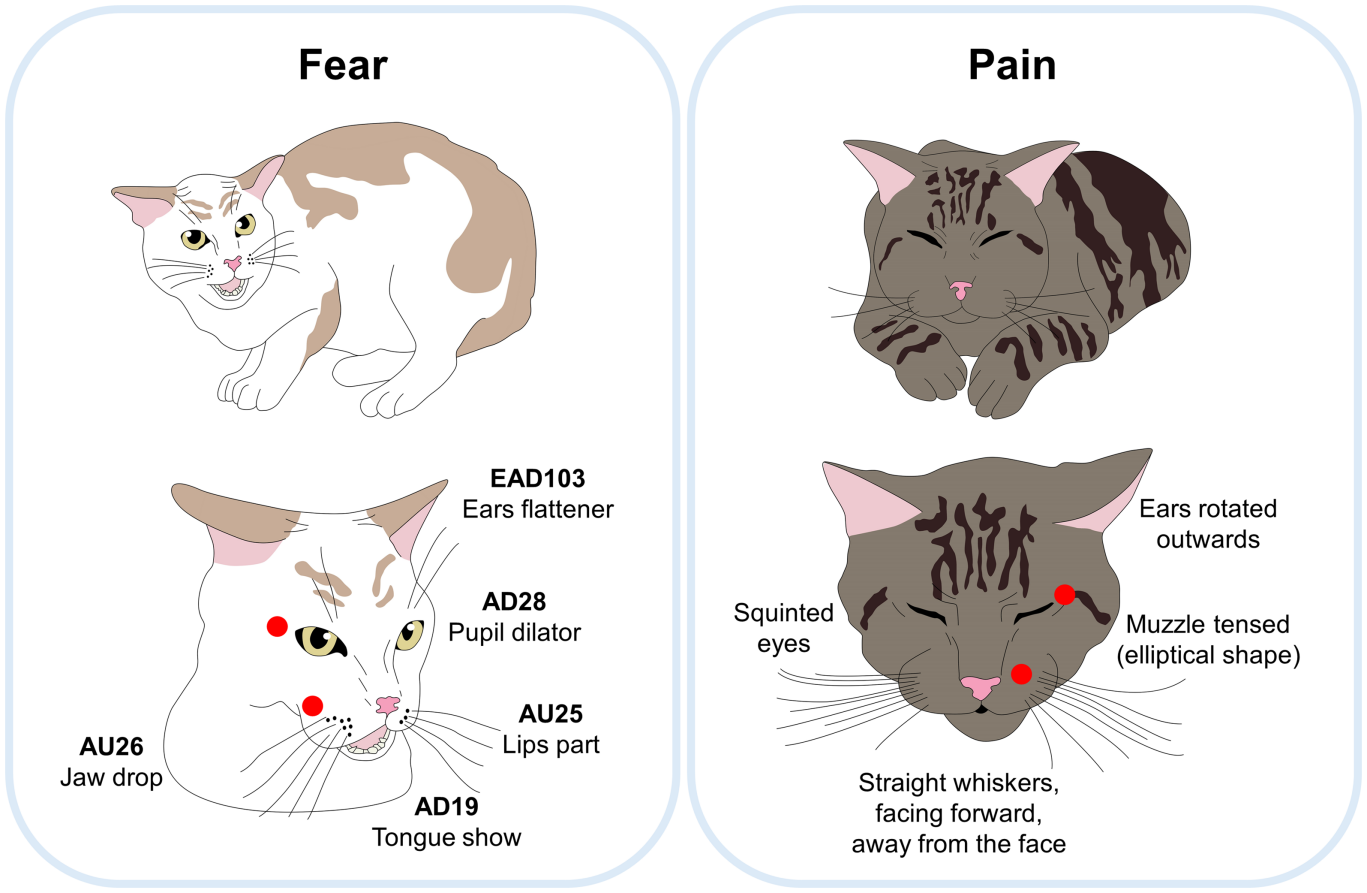
Comparison between a feline facial expression of fear and pain. Although both facial expressions use the FAU EAD103 (ear flattener), the main differences between both are marked with red circles. When cats feel fear, their eyes are kept wide open with marked pupil dilation, and their lips open (AU25). In contrast, when cats feel pain, the eyes and muzzle are obviously squinting, and muzzle with an elliptical shape without opening the mouth.

The authors observed an impact on facial expressions during the interspecific interaction that modified facial actions such as half blinking (AU 47), ears rotated and backward (EAD103 – EAD104), whiskers raised (AU200), yowling/growling (AD50), among others, replaced by eyes are directed upwards (AD63), ears flattened (EAD103) and lip corners retracted (AU12), which was associated with a social–emotional context, and illustrates the solidity of facial expressions as a tool to communicate the internal state of the animal (Figure 5). In addition to this, specifically in a species that retains characteristics of independence and self-sufficiency, such as the domestic cat, non-invasive monitoring optimizes its safety and well-being.

### 6.4 Updating FACS in equine

EquiFACS is one of the coding systems that has received the most attention due to the importance of horses as draught and companion animals. Moreover, the close relationship between humans and horses has increased the interest in recognizing emotional changes to improve the human-animal bond. In EquiFACS, Wathan et al. (29) described 17 AUs, with more movements than in humans or dogs.

**Figure 6:**
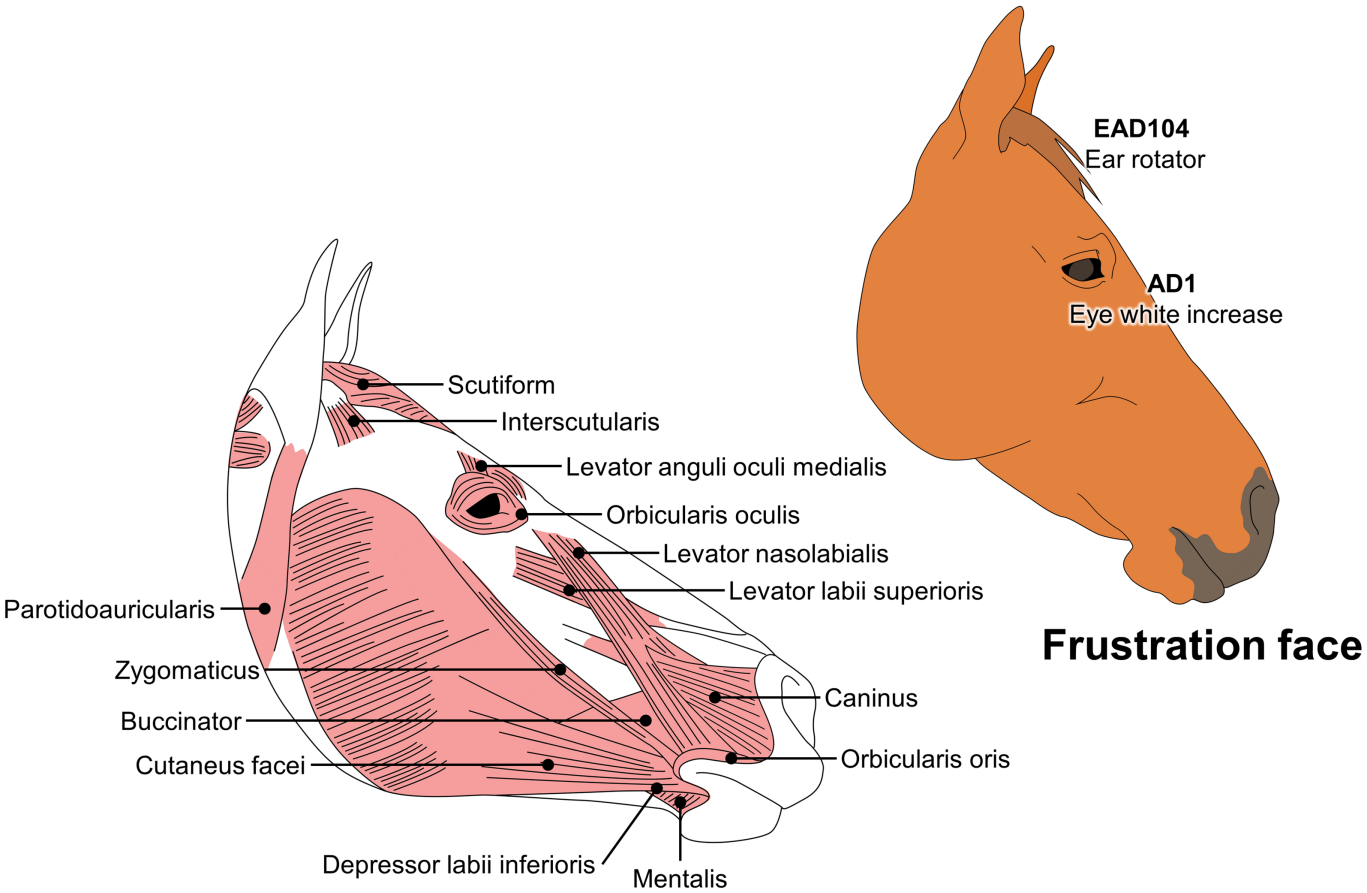
Codification of a frustration face in horses, according to the EquiFACS.

EquiFACS has been used to evaluate the responses of horses during social isolation and transport. Lundblad et al. (129) have reported facial changes and physiological alterations such asincreased heart rate. Similarly, Ricci-Bonot and Mills (155) identified potential facial markers of emotional states in 31 horses. They observed that frustration scenarios elicited changes in facial expression such as eye white increase (AD1), ear rotator (EAD104), and biting feeder, as well as a blink (AU145), nostril lift (AUH13), tongue show (AD19), chewing (AD81), and licking feeder. The authors concluded that these changes could be regarded as an equine facial expression of frustration, a valuable tool for identifying distinct facial movements during specific emotional states. For example, characteristic movements observed during frustration differ from those observed in an equine pain face (Figure 6).

## 7 Relationship between emotions, facial expression, and FACS

The neural networks that integrate both negative and positive emotions include the amygdala activation, and the contribution of sensory organs (e.g., retina, cochlea, or skin) to transduce and transmit the stimuli (156–162). Neurotransmitters such as dopamine or serotonin are closely related to reward systems in the brain, eliciting positive emotions (163). It is suggested that basic emotions are programmed to manifest through facial expressions as a non-verbal communication pathway (42). In the first instance, it must be established that as a standardization measure, FACS has been adapted to multiple species, which, through deep anatomical-functional analysis compile each of the possible biomechanical adjustments of the muscles (AUs) (16, 26, 28, 29).

As previously mentioned, brain circuits led by the amygdala coordinate the emotional response, including the movement of the facial muscles (1, 12, 164). The mimetic muscles of animals are innervated by branches of the facial nerve (VII). Studies in non-human primates (*Macaca fasciularis* and *M. fuscata*) have shown that the facial nerve is subdivided into four nuclear subdivisions. They comprise the medial, lateral, dorsomedial, and intermediate subnuclei and are responsible for basic musculotopic organization. For example, the medial subnucleus gives rise to axons that innervate the auricular and ocular musculature and the platysma; the lateral subnucleus innervates the upper lip and lower lip muscles; the intermediate portion deploys axons to the *Orbicularis oculi* and *Frontalis* muscles, and finally, the dorsal reaches the *Frontalis* muscle (165, 166).

However, the topographic limits of the subnuclei are unequal between species because it has been observed that specifically, the lateral and medial subnuclei of the VII nerve have different topographic limits, which allows variations in neuronal volume. These anatomical adjustments depend on the degree of participation or size of each muscle group. That is, in those species that frequently resort to facial expressions that involve the vibrissae, such as rats, mice, and opossums, a predominance of the subdivisions that control the movement of the nasolabial muscles is illustrated. For example, in mice, 43% of VII neurons are housed in the lateral subnucleus, which was positively correlated with the muscle volume of the nasolabial region. This tells us that there is a correspondence of magnitude between muscle fibers and the number of nervous structures that coordinate them (167).

On the other hand, cortical projections directed to the facial sub-nuclei have been discovered. For example, it was found that in Rhesus monkeys the primary motor cortex (M1), the caudal cingulate motor cortex (M4), and the ventral lateral premotor cortex (LPMCv) innervate the perioral musculature, while the supplementary motor area (M2) innervates the auricular musculature, and the rostral cingulate motor area (M3) the ocular muscles (165). However, direct synapse of cortical neurons with the VII nerve has only been reported in humans and non-human primates (166). In other species such as possums (*Didelphis marsupialis virginiana*) (168), armadillos (*Dasypus novemcintus*) (169), goats (170) and rats (*Rattus rattus*) (171), there is a traced pathway from the pyramidal tract to the parvocellular reticular formation close to VIIl.

Regardless of the origin, an emotional connection between corticofacial projections stands out due to their origin in limbic regions (166). A useful example is the recent study by Kunz et al. (172), where the brain mechanisms that control changes in facial expressions in humans subjected to affective experiences such as pain, were assessed using FACS. It was reported that brain activation of M2, M1, M3, and M4 increased during the painful stimulus, simultaneous with the manifestation of AU4 (brow lowerer) (*corrugator muscle*), AU6_7 (*orbicularis oculi muscle*), AU9_10 (*levator muscle*), AU 25_26_27 (*orbicularis oris muscle*); which relates to Krippl et al. (173) who tracked the neural correlates of voluntary facial movements in humans, such as AU1 + 2 (brow raiser), AU4 (brow lowerer), AU12 (lip corner puller) and AU24 (lip presser); observing greater activity in M1, M2, and LPMCv. Based on this evidence, it is possible to establish that during the emotional experience, the activity of higher emotional centers is synchronized with the M1 and subnucleotides of the facial cranial nerve.

Nerve endings from higher emotional centers also flow into the VII nerve. A clear reaction is the orofacial affective expression of ‘liking’ (AU5, AD37, AU 16–17, AD68, etc.) in human neonates and homologies in rats, great apes, and monkeys. Brain mapping has shown that the activation of antagonistic hedonic hotspots and negative aversive (‘disgust’) reactions is related to the nucleus accumbens, the ventral pallidum, the insular cortex, and orbitofrontal cortex. The fact that such expressions are manifested in human newborns suggests a conserved function to ensure effective non-verbal communication (67). These findings agree with other species such as horses, which exhibit nostril lift (AUH13), tongue show (AD19), and chewing (AD81) for periods of frustration due to the absence of food (155). Likewise, dogs subjected to denial access to food reward expressed greater predominance of lip parting (AU25), jaw drop (AU26), and nose licking (AD137) (28).

Involuntary facial expressions are unconscious and possibly governed by emotional and social contexts, so the dynamics between brain systems could direct facial expressions. Accordingly, the presence of efferent endings of amygdala origin to portions of the motor area has been illustrated. For example, through neural photographic representations of humans, the coordinated electrical charge between LPMCv, M2, amygdala, cerebellum, and facial nerve nuclei was observed during the sensation of opposite emotions such as positive taste ‘liking’ versus negative ‘disgust’ expressions (174). There is stronger evidence that intracerebral electrical stimulation (EEI) of the amygdala causes facial expressions of fear (muscle contraction of *medial frontalis* and *corrugator supercilii*) (AU109 + 110, AU200) in humans and mice (175). These facts suggest that, although involuntary, facial expressions are commanded from the brainstem, and cannot be considered simple reflexes, but rather carefully analyzed and selected indications by structures of the forebrain with higher hierarchical rank. Other points that add evidence are the connections with areas such as the thalamus that joins axonal projections from its ventral portion to the insular cortex (176) or the PAG that houses axonal endings of sensory, motor, and limbic structures. Therefore, it is related to mechanisms such as pain and fear, representing a reference point within the macrocircuits of emotional management (62).

In a simplified way, all those peripheral stimuli received through structures such as nociceptors (101, 177), tactile C fibers at the cutaneous level (162) or chemoreceptors at the upper gastrointestinal level (178) enter the spine through afferent endings to connect with nuclei that direct them to the limbic center and, trigger an activation response in the motor cortex that is conducted through the facial nerve and culminates in muscle groups of the face. For example, Furgala et al. (179) observed an increase in the manifestation of ear retraction and flattening in domestic cats (at least 8–15 individuals) subjected to prerecorded auditory stimuli such as the vocalization of a dog. It is possible to relate it to the neural route of stimuli of auditory origin, which are transmitted from the cochlea to different subregions of the thalamus and the auditory cortex that flow into the perirhinal cortex to directly inform the amygdaloid complex (90, 180) and possibly activate the M2 and M3 sections of the motor cortex that run through the brainstem to the leads of the medial subnucleus of the facial nerve (174) and cause the contraction of muscles such as *auricularis superior, abductor auris longus, abductor auris brevis, levator auris longus* to manifest EAD103 (flattening of ears) and EAD106 (ears drawn back).

Regarding inter-brain communication, higher centers have revealed multiple means of connection during emotional management. For example, it is known that the nucleus accumbens receives direct dopaminergic projections from the brainstem. Some studies in rats showed the relevance of this system in motivating the first positive hedonic “liking” (181). Therefore, the dopaminergic system can promote emotional mechanisms at the brain level. A dense population of neurons has been found in the amygdala that express dopaminergic receptors activated mainly during pleasant stimuli such as rewards (67). On the other hand, in neurological disorders such as Parkinson’s, dopaminergic depletion is exhibited in relation to the alteration in facial expressions, and it has been shown that the administration of dopamine increases the presentation and speed of facial movements in patients with Parkinson’s (182). Following recent findings in newborn humans, they showed that the compromise of fetal development absent the manifestation of facial movements such as mouthing and blinking. The authors associate this adjustment with restricted maturation of the central dopamine system. Therefore, it can be inferred that the dopaminergic system is involved in the modulation of facial expressions and emotional management.

Additionally, it has been shown that the facial motor nucleus maintains afferent connections with additional systems such as the serotonergic (5-HT). This is suggested to participate during emotional management since in studies of mice under fear conditioning, an extracellular release of 5-HT was observed at the basolateral level of the amygdala and prefrontal cortex (183). In this way, within the emotional mechanisms that control facial expressions, it is possible to include the serotonergic system. LeDoux et al. (184), analyzed the modulation of the serotonergic system on specific facial action units such as blink reflex (AU45) and blepharospasm in domestic cats (*Felis silvestris catus*) and primates (*Macaca mulatta*). Through the administration of the type-2 serotonin receptor (5-HT2) antagonist in the facial nucleus of the individuals, they were able to observe an increase in the frequency and speed of these facial actions, so they concluded that the innervation that predominantly expresses 5-HT reflects a relevant behavioral function in facial redesigns. Based on the evidence, it can be seen that the nucleus VII preserves independent axonal derivations in its structure that facilitate adaptive behaviors of an emotional nature.

In conclusion, the mechanism of action that promotes the relationship between emotions and facial expressions involves coordination between emotional management centers, primary motor cortex, subnuclei of VII, and facial muscles. In addition, this communication is optimized by molecular means that are activated or deactivated according to the valence of the emotion. Despite the anatomical differences between species, homogeneity can be established under the anatomical and functional constants of the brain.

## 8 Future directions

To date, only eight validated animal FACS have been published. However, the study and interest in facial expression has focused on other species, particularly in domestic animals such as cattle, sheep, or pigs (20, 185–187). For example, Lambert and Carder (188) summarized AUs in cattle during positive and negative emotions, where an upright ear posture is associated with excitement and a forward-facing ear posture is related to frustration. Likewise, de Oliveira and Keeling (186) associated body posture (e.g., neck and tail position) with certain facial movements including ear posture. It was found that, during brushing, the ears were asymmetrical and faced backward. In contrast, during negative contexts (e.g., queuing to enter an automatic milking system), the ears were axial and faced forward. Although these facial ethograms have been identified, there are no AnimalFACS for the species, and additional research must consider the AUs to codify each facial expression similarly to humans. Likewise, most research has focused on pain-associated AUs in laboratory animals such as rodents, rabbits, and ferrets. Nonetheless, limited information has been published regarding the changes in facial expression that animals might display during agonistic interactions or playtime.

According to the presented studies, facial expressions can help recognize an animal’s emotional state; however, important research gaps still need to be evaluated. One of these is the need to characterize the facial movements of animals under positive emotional conditions such as pleasure. Most reports indicate changes in the facial expression of animals exposed to negative conditions such as fear, frustration, and anger (127, 129, 189).

Current approaches focusing on animal welfare aim to not only identify negative contexts but also promote positive ones. Therefore, associating certain AUs and facial expressions could help to recognize which events indicate good welfare and, thus, good quality of life (190).

Additionally, the brain mechanisms that control facial expressions during emotional management are partially revealed, since although the regions of greatest activation have been discovered through lesion, mapping, or inactivation assays, the complete neuronal pathway and the chemical mediators involved are still unknown (165, 166, 191). In addition to this, the anatomical difference between species is evident, so these distinctions and their functional effect must also be pointed out (166, 167).

## 9 Conclusion

Facial expressions are considered a manifestation of an emotion regulated by specific muscles. Facial movements and even mimetic muscles depend on the species and research has not been performed on all mammals. The fact that nonhuman animals cannot verbally express the emotional valence of certain events limits the full understanding of animal emotion. However, in recent years, it has been accepted in several parts of the world that animals are sentient beings, capable of experiencing affective states such as pleasure, fear, and anxiety. Emotional stimuli, both positive and negative, trigger a series of physiological and behavioral responses that allow an important and indispensable analysis in the field of animal welfare.

AnimalFACS is a system that objectively codifies facial movements according to their muscular basis. Not all species have validated FACS; however, characterizing each facial expression using AUs in several contexts that might have a negative or positive affective response is an approach to understanding emotion and nonverbal communication in nonhuman animals.
